# Microbiologically Induced Carbonate Precipitation in the Restoration and Conservation of Cultural Heritage Materials

**DOI:** 10.3390/molecules25235499

**Published:** 2020-11-24

**Authors:** Erick Ortega-Villamagua, Marco Gudiño-Gomezjurado, Alex Palma-Cando

**Affiliations:** 1 Grupo de Investigación Aplicada en Materiales y Procesos (GIAMP), School of Chemical Sciences and Engineering, Yachay Tech University, Hda. San José s/n y Proyecto Yachay, Urcuquí 100119, Ecuador; erick.ortega@yachaytech.edu.ec; 2 School of Biological Sciences and Engineering, Yachay Tech University, Hda. San José s/n y Proyecto Yachay, Urcuquí 100119, Ecuador; mgudino@yachaytech.edu.ec

**Keywords:** microbiologically induced carbonate precipitation (MICP), conservation, restoration, cultural heritage, calcium carbonate

## Abstract

Microbiologically induced carbonate precipitation (MICP) is a well-known biogeochemical process that allows the formation of calcium carbonate deposits in the extracellular environment. The high concentration of carbonate and calcium ions on the bacterial surface, which serves as nucleation sites, promotes the calcium carbonate precipitation filling and binding deteriorated materials. Historic buildings and artwork, especially those present in open sites, are susceptible to enhanced weathering resulting from environmental agents, interaction with physical-chemical pollutants, and living organisms, among others. In this work, some published variations of a novel and ecological surface treatment of heritage structures based on MICP are presented and compared. This method has shown to be successful as a restoration, consolidation, and conservation tool for improvement of mechanical properties and prevention of unwanted gas and fluid migration from historical materials. The treatment has revealed best results on porous media matrixes; nevertheless, it can also be applied on soil, marble, concrete, clay, rocks, and limestone. MICP is proposed as a potentially safe and powerful procedure for efficient conservation of worldwide heritage structures.

## 1. Introduction

Minerals precipitation by living organisms activity, so-called biomineralization, is a process that occurs from bacteria to chordates [1]. This mineral formation occurs through two different processes. The first takes place in various animals in a process where the organism produces an organic framework to introduce ions for further crystallization and growth mediated by an organic matrix. The second is distinguished by massive intracellular and/or extracellular mineral formation commonly in the form of teeth, skeletons, shells, etc. [2,3]. The precipitation of minerals by microorganisms is obtained by the modification of the local environment as a result of the metabolites release. This releasing of molecules increases pH and elevates the supersaturation, resulting in the precipitation of minerals. In addition, some macromolecules and cell structures can act as heterogeneous crystallization nuclei, inducing the precipitation [4,5]. The different type of minerals that bacteria are able to produce includes nitrates, silicates, calcium oxalates, halides, apatite, gypsum, oxides, phosphates, and calcium carbonate [4,6]. The process of calcium carbonate precipitation is present in nature, commonly in marine environments, freshwater, and soil (e.g., solid surfaces) [7,8]. Calcium carbonate may precipitate through the attachment of the calcium ions to the microbial cell walls or to the extracellular polymeric substances, which act as crystal nucleation sites [9,10]. Depending on the cell surface properties of bacteria, especially proteins and extracellular polymeric substances, the morphology and mineralogy of calcium carbonate can be varied, e.g., rhombohedral (calcite), hexagonal (vaterite), or needle-like crystal (aragonite) [9], being calcite the most stable molecular structure [11]. Microbiologically induced carbonate precipitation (MICP) is mainly driven by factors, such as pH, Ca^2+^ concentration, dissolved inorganic carbon concentration, and availability of nucleation sites [12]. Microorganisms use different metabolic pathways to induce CaCO_3_ precipitation; however, this process is not entirely defined yet. Genetics and physiology involved in the process are quite a challenge to understand [13]. Some of the metabolic pathways involved in CaCO_3_ precipitation are anaerobic sulfide oxidation, photosynthesis, methane oxidation, ammonification, denitrification, sulfate reduction, and ureolysis [10].

**Figure 1:**
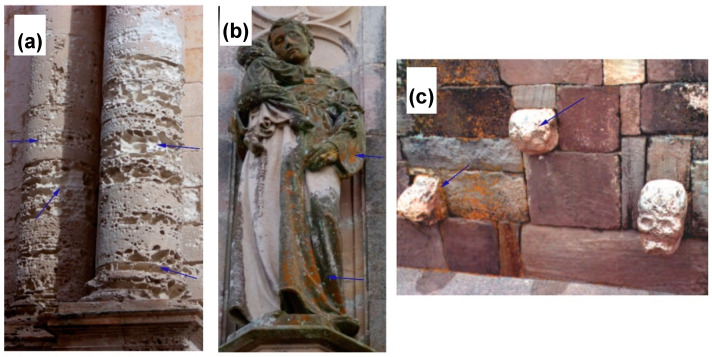
Degraded historic buildings by (**a**) a massive salt weathering in building stone, and (**b**) staining and microbial growth. Modified with permission from [27]. 2017, Consejo Superior de Investigaciones Científicas (CSIC). (**c**) Skull shape, completely lost with the presence of orange lichen. Modified with permission from [16].

On the other hand, chemical, physical, biological, and anthropogenic factors are the principal perpetrators of monumental stone decay. Architectural structures and monuments begin to weaken through progressive matrix dissolution and porosity increase [14], resulting in deteriorative effects, such as inclination and discoloring [15], water retention, growth of heterotrophic and higher organisms, patinas formation, corrosion, alkaline dissolution, among others (see Figure 1) [16]. Due to the common social and historical value of these structures, conservation and restoration approaches should be addressed by scientists and conservators in a joint interdisciplinary work [15]. Conventional organic and inorganic conservation treatments show several drawbacks. Synthetic resins (e.g., silane, epoxy, acrylic, polysiloxane) polymerize and plug the stone pores, retaining water and accelerating the internal degradation [16]. External protecting coats tend to deteriorate, peels off, and requires maintenance [17], while additional noxious solvents might be released by decomposition [18]. Limewater treatments and inorganic solutions based on Ba(OH)_2_ [19] and Ca(OH)_2_ [20] usually lead to non-consolidating calcite superficial aggregation [15,21]. These problems have motivated researchers to seek alternative methodologies. Bacterial calcium carbonate precipitation was proposed as a method for the restoration of calcareous stones as one of the most vulnerable materials against deterioration. This treatment, patented in 1990 (expired in 2010), aims for the production of a superficial coating of calcium carbonate by using living cultures of bacterial strains [22]. Calcium carbonate is involved in the restoration process through the biological healing, that is, the production of calcium carbonate commonly through urease producer bacteria (10^6^–10^8^ colony-forming unit (CFU)), in an aerobic environment and in the presence of a calcium source [11,23,24]. The carbonatogenesis helps the concrete micro-cracks sealing, avoiding the penetration of water into the rock or cement matrices. Resistance and strength of these materials [25,26] get boosted under an energy-efficient mechanism in an eco-friendly way [9,23]. This methodology has already been proved to reduce stone porosity, resulting in more consolidated structures [14,21].

In this short review, we summarize techniques and strategies conducted by researchers in the field of bacterially induced CaCO_3_ precipitation for the conservation of heritage materials. A short section of biotic carbonate formation is presented, followed by a description of the most common mechanisms of bacterial precipitation through ureolysis, tests in vitro and in situ with their respective results for the conservation of calcareous historical stones, and finally recommendations and limiting factors for this type of treatment. Microbiologically induced carbonate precipitation (MICP) has been reviewed for its use in biotechnology [10], engineered applications [28], sand treatment [29], and environmental problems [23], but, as far as we know, until now, a review with priority in its application for conservation and restoration in cultural and historical heritage has not been addressed. The major purpose of this review is to present MICP as one powerful consolidation and restoration technique for historical building materials. There is not a consensus on the optimal conditions for the application of this type of treatment; therefore, some branches of science (chemistry, biology, historical restoration, materials science and engineering, geology) could be interested in the standardization and optimization of this methodology.

## 2. Biogenic Precipitation of Calcium Carbonates

Calcium carbonate comprises more than 4% of the earth’s crust. This compound is found in chalk, marble, travertine, tufa and even is the principal component of shells and pearls [9]. Calcium carbonate biomineralization can occur by two different mechanisms based on the degree of microbiological control, named as microbiologically controlled carbonate precipitation (MCCP) and microbiologically induced carbonate precipitation [30]. In MCCP, the cells of the organisms designate a site for mineral formation, such as polymerized macromolecules, membranes, or vesicles, after that, ions are imported in a regulated sequence. Crystal nucleation and growth control induced by organic matrices differ from different phyla, but, in general, occur over solid surfaces, such as membranes, macromolecular substrates, and extracellular polymeric substances (EPS) [2,3,30]. Mineral particles are formed intracellularly under metabolic and genetic control, leading to specialized structures like exoskeletons, teeth, and shells. The formation of these structures works independently from environmental conditions [2,31]. In comparison, MICP is regulated by the combined physiological activities of microorganisms. This process is carried out in open environments; therefore, external physical-chemical parameters play an important role in the type of mineral produced [30]. MICP usually occurs in the extracellular environment, commonly driving to the mineralization of the proper bacterial cells. One of the most accepted hypotheses for CaCO_3_ precipitation considers that calcium ions are not used by microbial metabolism; instead, they are aggregated and crystallized in the cell surface using EPS as nuclei for crystallization [5,32,33,34]. In general, the CaCO_3_ precipitation rate is a linear function dependent on Ca^2+^ and ${\mathrm{CO}}_{3}^{2-}$ ions concentration product, therefore, following second-order kinetics or pseudo-first-order kinetics if there is an excess of one of the reagents [35]. Bacteria can influence the reachable saturation and rate of carbonate precipitation by controlling the CaCO_3_ crystal polymorph produced. Supersaturation (S) is only reached when the solubility product (K_sp_) is exceeded by the concentration of [Ca^2+^] and $\left[{\mathrm{CO}}_{3}^{2-}\right]$. Precipitation of CaCO_3_ is favored with higher supersaturation level and defined by Equation (1) [35]:

(1) $$S=\frac{\left[{\mathrm{Ca}}^{2+}][{\mathrm{CO}}_{3}^{2-}\right]}{K_{\mathrm{sp}}}$$

Calcium carbonate can precipitate as any of the six polymorphs. Listed in increasing thermodynamic stability, CaCO_3_ polymorphs are (i) amorphous calcium carbonate, (ii) calcium carbonate monohydrate, (iii) calcium carbonate hexahydrate, (iv) vaterite, (v) aragonite, and (vi) calcite [36]. Although researches have reported the presence of almost all polymorphs in CaCO_3_ bioprecipitation, calcite and vaterite are the most common precipitates [37]. Some parameters controlling the type of precipitated polymorphs are the medium supersaturation level, presence of glycoproteins and amino acids, the solubility of the possible phases [36], the specific bacterial strain, specific proteins present in EPS, dissolved organic carbon [37], abiotic factors, complex interactions associated with organic molecules, the order in the addition of reactants, among others [38]. This extended variety of possible parameters, which influence the morphology and the formation of polymorphs by bacteria, is the main reason for a no consensus on the main mechanism that affects the biomineralization of polymorphs. The extracellular calcium carbonate synthesis occurs by autotrophic and heterotrophic pathways. Algae and cyanobacteria are responsible for the autotrophic pathway through fixing carbon dioxide to carbonate by means of (i) anoxygenic photosynthesis, (ii) non-methylotrophic methanogenesis, and (iii) oxygenic photosynthesis [9,39]. On the other hand, *Arthrobacter*, *Bacillus*, and *Rhodococcus* have been described as microorganisms capable of employing organic salts as an energy source, producing carbonate minerals, such as magnesium carbonate or calcium carbonate, in caves, marines, lakes, and soils [11]. In the presence of a calcium source, bacteria of the genera *Bacillus*, *Lysinibacillus*, and *Sporosarcina* can produce calcium carbonate through urea hydrolysis. Among these genera, the most common species described as healing crack microorganisms are *Bacillus amyloliquefaciens*, *Bacillus cereus, Lysinibacillus sphaericus*, and *Sporosarcina pasteurii* [11].

## 3. Bacterial CaCO_3_ Precipitation Through Ureolysis

**Figure 2:**
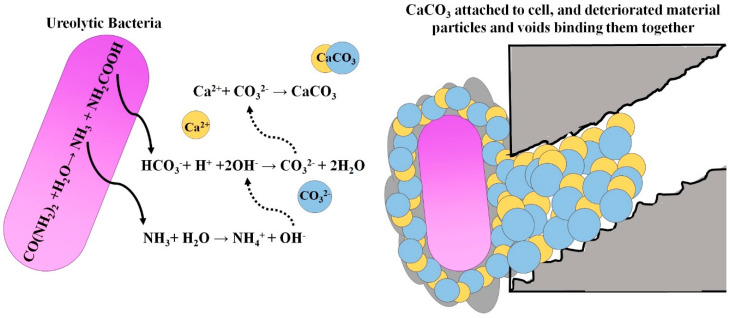
A general overview of chemical processes involved in ureolytic calcium carbonate precipitation.

The most common method for CaCO_3_ bioprecipitation is urea hydrolysis [10]. When bacteria with ureasic activity are in the presence of a medium that contains urea (CO(NH)_2_)_2_ and Ca^2+^ ions, they might produce calcium carbonate precipitates. Ureolytic bacteria can produce urease enzyme. Bacterial urease (urea amidohydrolase E.C.3.5.1.5) is a multi-subunit nickel metalloenzyme composed of two (αβ) or three (αβγ) subunits [40]. Jabri et al. described the crystal structure of *Klebsiella aerogenes* urease, which consists of two nickel atoms linked by a carbamate group [41]. This metallic center acts as a catalytic center for the reaction of urea to carbamic acid and ammonia [42]. An increase in pH by ${\mathrm{NH}}_{4}^{+}$ production fosters the CaCO_3_ precipitation. The catalytic activity of this kind of enzymes depends on the temperature and the bacterial species. For example, Anbu et al. reported the optimum temperature for most ureases ranging from 20 °C to 37 °C [23]. On the other hand, the enzymatic activity of these enzymes may differ among the species or even between the strains of the same species. For instance, the enzymatic activity of *Bacillus megaterium* urease decreases at high temperature, while at low temperature, surpasses the enzymatic activity of *Sporosarcina pasteurii* [43]. In fact, the genera *Bacillus and Sporosarcina* are known for their high production levels of urease, which are the most frequently used ureolytic bacteria for biotechnological applications [10,44,45]. Another factor that defines the urease activity is the urea and calcium concentrations [46]. De Muynck et al. reported that the best calcite production was achieved with 0.25 M and 0.5 M of calcium chloride and urea, respectively [47]. The availability of nucleation sites is another key factor that governs the rate of precipitation [36]. During the bioprecipitation process, particles in suspension [48], dust particles [36], and bacteria themselves serve as active sites for calcite nucleation [49]. The bacterial cell surface is typically negatively charged; hence, it is able to attach divalent cations like Ca^2+^ or Mg^2+^ [50]. In more detail, the mechanism of precipitation begins with the urea hydrolysis, producing carbamic acid and ammonia (Equation (2)). Spontaneous decomposition of carbamic acid produces carbonic acid and ammonia (Equation (3)). Ammonia and carbonic acid equilibrate in their protonated and deprotonated form in the aqueous medium, modifying the pH (Equations (4) and (5)). Actually, ammonia formation increases the pH up to 9.2, which favors the calcium carbonate bio-mineralization [9,11,43]. Reaction continues towards calcium carbonate precipitation by bonding Ca^2+^ ions to the bacteria cell surface (Equation (6)). The high concentration of carbonate and calcium ions on the bacterial surface, which serves as nucleation sites, promotes calcium carbonate precipitation (Equations (7) and (8)) that could bind and consolidate deteriorated materials in historical structures (see Figure 2) [35,51,52].

(2) $$\mathrm{CO}{\left({\mathrm{NH}}_{2}\right)}_{2}+H_{2}O\vec{\;(\mathrm{urease})\;}{\mathrm{NH}}_{3}+{\mathrm{NH}}_{2}\mathrm{COOH}$$

(3) $${\mathrm{NH}}_{2}\mathrm{COOH}+H_{2}O\to {\mathrm{NH}}_{3}+H_{2}{\mathrm{CO}}_{3}$$

(4) $$H_{2}{\mathrm{CO}}_{3}⇌{\mathrm{HCO}}_{3}^{-}+H^{+}$$

(5) $$2NH_{3}+2H_{2}O⇌2NH_{4}^{+}+2OH^{-}$$

(6) $${\mathrm{Ca}}^{2+}+\mathrm{cell}\to \mathrm{cell}-{\mathrm{Ca}}^{2+}$$

(7) $${\mathrm{HCO}}_{3}^{-}+H^{+}+2{\mathrm{OH}}^{-}⇌{\mathrm{CO}}_{3}^{2-}+2H_{2}O$$

(8) $$\mathrm{cell}-{\mathrm{Ca}}^{2+}+{\mathrm{CO}}_{3}^{2-}\to \mathrm{cell}-{\mathrm{CaCO}}_{3}↓$$

## 4. Microbiologically Induced Carbonate Precipitation on Solid Samples in Laboratory

Microbiologically induced carbonate precipitation is a controlled process that can be used for different applications, such as improvement of concrete mechanical properties and self-healing of cracks [53], heavy metals removal [54], sand and clay biocementation [55,56,57,58,59], dust suppression, radionuclide remediation [28], CO_2_ sequestration [60], and conservation and restoration of historical and cultural objects [61]. In 1973, Boquet et al. isolated 210 microorganisms from the soil, which precipitate calcite crystals in a suitable environment [62]. Since this pioneering work, various research groups have been studying and testing microbial carbonate precipitation onto surfaces, commonly stone or marble slabs, changing conditions, such as calcifying bacteria strain, metabolic pathways, nutrient media, pH, among others. Calcium carbonate bioprecipitation is gaining interest among researchers over the last decades for non-conventional applications [10].

**Figure 3:**
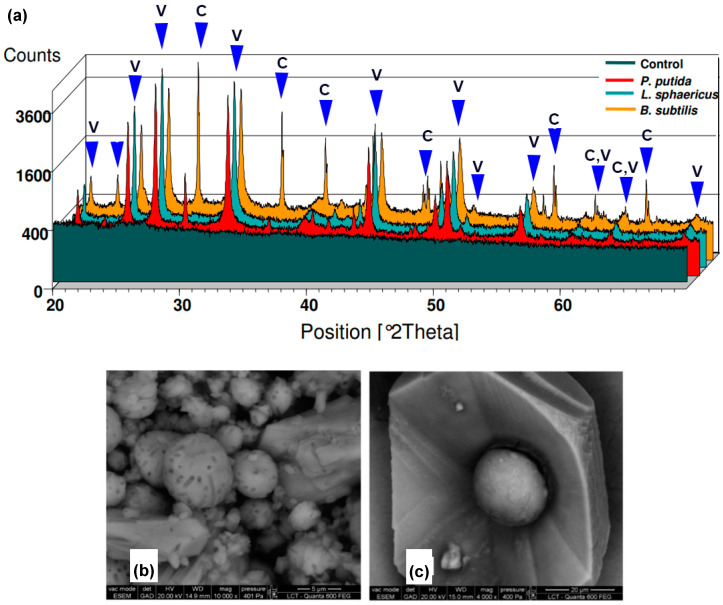
(**a**) The comparison of x-ray powder diffraction (XRD) patterns between control samples, *P. putida, L. sphaericus*, and *B. subtilis*. Environmental scanning electron microscopy (ESEM) images of (**b**) calcium carbonate crystals with bacterial presence evidence, and (**c**) spherical calcium carbonate within a rhombohedral and pinacoidal crystal. Adapted with permission from [7].

There are different studies about the implementation of new strategies to improve the yield of calcium carbonate production, allowing further applications in restoration and conservation. Shirakawa et al. compared the efficiency of three different bacterial strains (*Lysinibacillus sphaericus, Pseudomonas putida*, and *Bacillus subtilis*) for calcium carbonate precipitation [7]. Bacteria were inoculated in two different culture media with no pH adjustment, (i) modified B4 media with calcium acetate as Ca^2+^ source, and (ii) 295 media with calcium chloride as Ca^2+^ source. The samples were incubated in static and shaking conditions at 28 °C for 12 days. After a period of incubation, atomic absorption spectrometry [63] was used to measure the concentration of calcium ions remaining in the media as an indicator of calcium carbonate production. Bacteria in B4 modified the media-generated greater CaCO_3_ precipitation compared to bacteria in 295 media. Average calcium consumption in B4 modified media was 96% for *P. putida*, 74% for *L. sphaericus*, and 28% for *B. subtilis*, in comparison with sterile media. X-ray powder diffraction (XRD) [64] showed that *B. subtilis* and *P. putida* produced vaterite and calcite, while *L. sphaericus* produced the only vaterite in B4 modified media (see Figure 3a). Environmental scanning electron microscopy (ESEM) [65] showed, for all strains in shaking conditions, the production of smaller calcium carbonate crystals than static conditions. Rectangle, hemispherical, spherical, rhombohedral, and pinacoidal crystals were formed under these conditions (see Figure 3c). Higher magnification showed holes inside crystals, which coincided with the cell size of *Bacillus* strains, pointing out for bacteria cell surface as nucleation sites (see Figure 3b). This study proved for the first time that static and shaking conditions at the same temperature could alter the shape and size of the CaCO_3_ bioprecipitated crystals.

Schwantes-Cezario et al. tested *B. subtilis* for calcium carbonate bioprecipitation [13]. *B. cereus* and *E. coli* were used as a positive and negative control, respectively. B4 was used as culture media adjusted at different pH conditions (non-buffered, basic buffered at pH = 8.2, and neutral buffered at pH = 7.0). Bacteria were incubated with constant agitation at 37 °C for 7 days. After incubation, calcium carbonate precipitation was only observed in non-buffered and basic conditions. Scanning electron microscopy (SEM) [66] images indicated the same crystal morphology for *B. subtilis* and *B. cereus* as rounded structures. Quantification of CaCO_3_ concentration exhibited no significant difference between *B. subtilis* $\left(\frac{1.325\;g\;{\mathrm{CaCO}}_{3}}{1\;\mathrm{mL}\;B4\;\mathrm{media}}\right)$ and *B. cereus* $\left(\frac{1.291\;g\;{\mathrm{CaCO}}_{3}}{1\;\mathrm{mL}\;B4\;\mathrm{media}}\right)$. Large biofilms were obtained, showing the potential to be used in crack filling purposes for increasing lifespan or preventing early deterioration. Similar results were reported by Páramo Aguilera et al., who found optimal biofilm formation by using *B. subtilis* and *B. cereus* bacterial strains [67].

Marvasi et al. showed that buffering B4 media at pH values of 7.3 and 8.2 influenced the mineral bioprecipitation [33]. After two weeks of incubation, crystal precipitation was observed from the 49 isolated strains at 39 °C. Moreover, 73% of the isolates were able to produce crystals at pH = 8.2, whereas media buffered to pH = 7.3 inhibited the crystal precipitation in 79% of the strains. Five strains of *Lysinibacillus sphaericus* (strain numbers 55,56,57,58,59) and one strain of *Bacillus lentus* (strain number 60) were tested for the carbonate bioprecipitation on Euville limestone surface by Dick et al. [68]. Ureolytic calcium carbonate precipitation was proposed to be followed using a medium that contains nutrient broth, sodium bicarbonate, urea, and CaCl_2_ as Ca^2+^ source. After 12 h of incubation for *B. lentus*, slow CaCO_3_ precipitation was shown, and hence deficient CaCO_3_ deposition. Samples with *L. sphaericus* strains (55,56,58) began precipitation after four hours of incubation. SEM images revealed the deposition of calcite crystals on the sample surface. Strain 58 showed a heterogeneous distribution of calcite crystal, whereas strain 59 produced rhombohedral homogenous calcite crystal deposition. Treated limestone surface with strains 57 and 59 showed a reduced capillary water absorption [69] within two days. The electrical charge at the shear plane (ζ-potential) [70] originated from (de)protonation or (de)complexation of surface molecules was linked to homogeneous surface limestone colonization. The highly negative ζ-potential at the surface of the bacteria cell and the positive ζ-potential of the limestone set appropriate conditions for bacterial colonization and precipitation. Bacterial strain colonization capacity depends on its negative *ζ*-potential. Strains 56, 57, and 59 presented the most negative ζ-potential at pH 9. These strains had a significant impact, decreasing limestone capillary absorption property.

**Figure 4:**
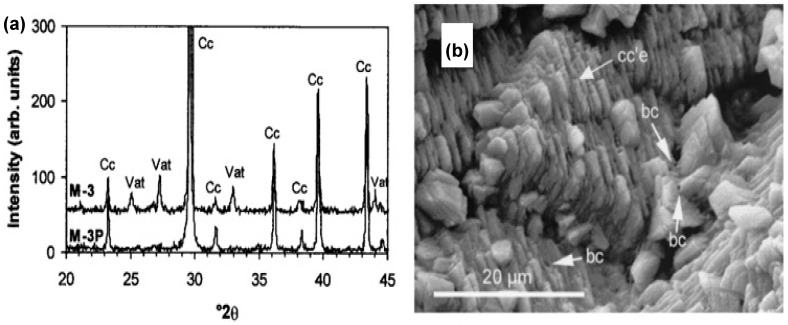
(**a**) XDR patterns of slabs subjected to microbiologically induced carbonate precipitation (MICP); (**b**) formed calcite crystals, developing epitaxially (cc’e) on pre-existing calcite crystals and showing preferred crystallographic orientation. Adapted with permission from [71] 2003, American Society for Microbiology.

Rodriguez-Navarro et al. used the theory behind bioprecipitation to test if this technique generates good results in the consolidation and protection of limestone slabs [71]. *Myxococcus xanthus* (strain number 422) was used as the calcifying bacteria in two different culture media of non-buffered M-3 (with Ca(CH_3_COO)_2_ as a source of Ca^2+^) and buffered M-3P (in phosphate buffer) at pH = 8. The experiment was carried out by using test tubes for small slabs (0.5 cm^3^) under shaking conditions and Erlenmeyers for larger slabs (5.63 cm^3^) under static conditions. Incubation was performed at 28 °C for 30 days; however, only 5 to 10 days were required to give high yields of the precipitates. Results showed that weight changes due to the precipitation of carbonates began around 2.5 days for small slabs and 5 days for larger ones. XRD showed the formation of calcite and vaterite, with calcite as the main precipitated phase (see Figure 4a). SEM analysis revealed sparitic and rhombohedra calcite crystals and needle-shaped vaterite crystals. Epitaxial growth was observed over the pre-existing crystals, resulting in the precipitation of carbonate, without blocking or plugging the pores in the slabs (see Figure 4b). Sonication removed the biofilm formed, although calcified bacteria cells and vaterite crystals were not removed in appreciable amounts for larger slabs. Slabs incubated in the M3-P media showed lower weight loss. Newly formed crystals were strongly added to the surface and were more resistant than the pre-existing ones.

**Figure 5:**
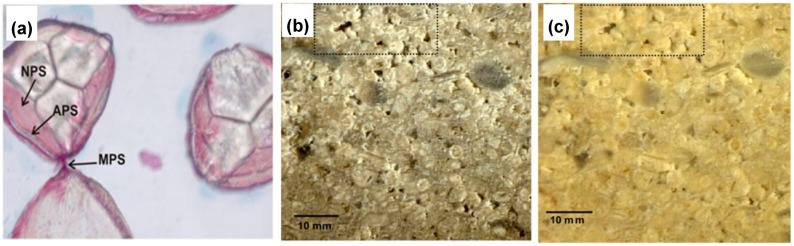
(**a**) Carbonate crystals precipitated by isolate B14 joined by a mixture of acid and neutral polysaccharides. Magnification ×200. NPS, neutral polysaccharides; APS, acid polysaccharides; MPS, mixture of neutral and acid polysaccharides. Isolate D2 on limestone (**b**) before treatment and (**c**) after MICP treatment. Dash line rectangles allow the visual comparison of pore size. Adapted with permission from [72] 2009, American Society for Microbiology.

In 2009, histological stains were used for the first-time by Zamarreño et al. to reveal polysaccharides surrounding carbonate crystals [72]. *Pseudomonas* (D2 and F2) and *Acinetobacter* (B14) strains were both isolated from freshwater. Bacterial strains were inoculated in B4 modified media before spreading over 500 µm thick limestone slides and incubated over three weeks at 30 °C. Bacterial distribution was determined on crystals and limestone slides by staining live bacteria with 5-cyano-2,3-ditolyl tetrazolium chloride (CTC) and polysaccharides with alcian blue–periodic acid-Schiff stain (AB-PAS). After the incubation period, B14 and D2 samples precipitated light and dark brown crystals, respectively, while the F2 sample precipitated light green crystals. The main polymorph distribution was calcite for D2 and F2 and vaterite for B14, showing spheroidal crystal morphology. Histological staining showed carbonate crystals surrounded by bacteria for sample F2, with a uniform distribution between the inner and outer core. Thick sections of B14 precipitates revealed the presence of polysaccharides in the center of the crystal. Moreover, stain revealed that multiple crystals were bonded by neutral and acid polysaccharides (see Figure 5a). Carbonate precipitates from sterile modified B4 media reduced the open pore area by 19%. On the other hand, carbonate deposits from inoculated media for B14, F2, and D2 samples reduced open pore area by 43%, 46%, and 49%, respectively (see Figure 5b,c). Bacterial presence at least duplicated the pore reduction area. After 330 days, no presence of viable cells was found for crystal precipitates from B14 and F2 samples, and only a few viable cells (~9 cell/mg) were found on crystals precipitated by isolate D2. These results contrast with the findings of Rodriguez-Navarro et al. [4], who reported almost the same consolidation without bacteria inoculation.

**Figure 6:**
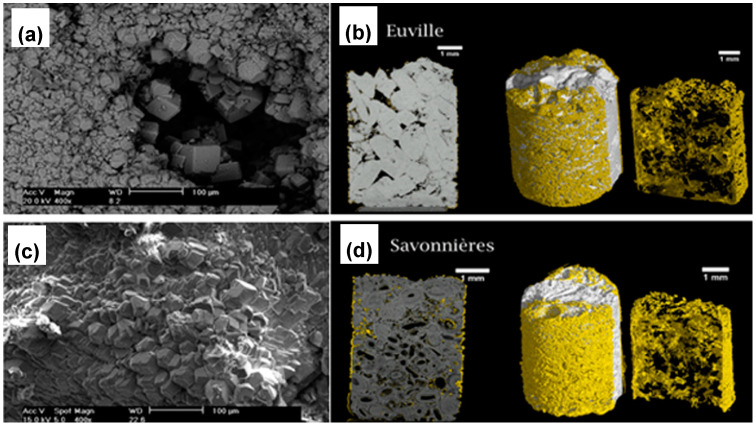
Scanning electron microscopy (SEM) images of MICP-treated limestone: (**a**) Aubigny and (**c**) Euville samples. 2D (left) and 3D (middle and right) microtomograph of MICP-treated limestone: (**b**) Euville and (**d**) Savoniers samples. Newly formed carbonate crystals are yellow. Adapted with permission from [73] 2011, American Society for Microbiology.

De Muynck et al. studied the effectiveness of the carbonatogenesis treatment and the difference in the protective performance between macro and microporous stones [73]. Five types of porous french limestone were treated: (i) Avesnes (32.10% porosity), (ii) Savonnières (30.90% porosity), (iii) Euville (17.24% porosity), (iv) Aubigny (14.12% porosity), and (v) Massangis (9.98% porosity). Several specimens were cut into small pieces in the form of prisms, cubes, or cylinders. Biodeposition treatment was divided into two steps. First, stone pieces were immersed in media containing yeast and urea (pH 9.45) inoculated with *L. sphaericus* culture under static and nonsterile conditions at 28 °C for 24 h. For the second step, stone pieces were immersed in a sterile medium containing urea and calcium chloride for four days. SEM micrographs revealed crystal precipitate variations in morphology and size (see Figure 6a,c). Energy-dispersive X-ray spectroscopy (EDX) [74] confirmed that newly precipitate crystals consisted of calcium carbonate. Depending on the type of stone, microtomographs showed large differences in the degree of coating and penetration depth of the biocoating (greater than 2 mm for Euville and Savonnières) (see Figure 6b,d). MICP treatment produced weight gain in all the stone samples, which was more noticeable in the most porous stones. Spectrophotometric analyses exhibited significant color changes, and the overall degree color change (∆E) values fluctuated between 14.6 and 7.3. Capillary water absorption tests showed, for all stones, the diminished rate of water uptake, with a 20 times reduction for Savonnières samples. Resistance to sonication tests showed around 50% less weight loss of MICP-treated stones compared to untreated ones. Cycles of sodium sulfate exposure revealed that MICP-treated stones increased the resistance to salt attack, which was more noticeable in the most porous stones. Treated Savonnières and Massangis limestones remained almost unaffected after 15 and 20 cycles of salt attack, respectively. MICP-treated stones displayed higher resistance to freezing and thawing cycles compared to untreated ones. It seems that the pore structure influences the coating distribution and production of newly formed crystals. Greater calcium carbonate protective effect and precipitation throughout stones with a large number of macropores is explained by cell size. The size of *L. sphaericus* lies between 1 µm to 4 µm, meaning that pores bigger than 2 µm of radius are necessary to obtain maximum microbial cell absorption.

**Figure 7:**
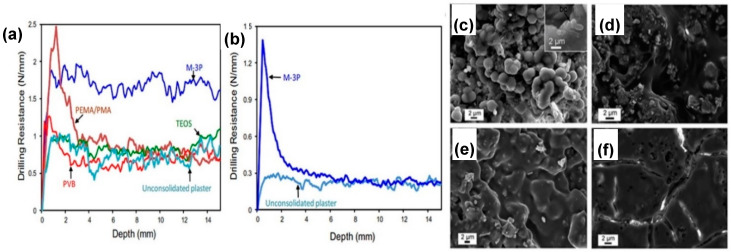
(**a**) Drilling resistance (DR) measured on gypsum plaster pieces, with an initial porosity of 29–38% and treated with three conventional consolidants and M-3P culture medium. (**b**) DR of untreated and bacterially treated gypsum plaster with an initial porosity of 48%. Values are averages of three to five drill holes. Samples treated with (**c**) M-3P, showing bacterial vaterite surrounded by EPS and bacterial cells (inset shows a magnification), (**d)** poly(ethyl methacrylate-co-methacrylate) (PEMA/PMA), (**e**); polyvinyl butyral (PVB), and (**f**) tetraethyl orthosilicate (TEOS). Adapted with permission from [75] 2014, Elsevier.

Limestone is not the unique material able to be treated via calcium carbonate biomineralization. Calcium sulfate dihydrate (CaSO_4_ 2H_2_O), known as gypsum, has been used since 12,000 BC as decorative or building material worldwide. Jroundi et al. reported carbonate bioprecipitation as a consolidant on archeological gypsum plaster [75]. In this study, fifteen historical gypsum pieces (dated from the fourteenth century) were obtained from “Alcazar Real de Guadalajara”, Spain. Calcifying bacterial treatment was carried out by spraying sterile M-3P nutrient media twice a day for 6 days to reactivate the bacterial strains already present in the gypsum samples. Conventional consolidants of tetraethyl orthosilicate (TEOS), polyvinyl butyral (PVB), and poly(ethyl methacrylate-co-methacrylate) (PEMA/PMA) were applied on gypsum pieces with a brush every 24 h for 7 days for comparison. After treatment, the drilling resistance (DR) test [76] was used for measuring the consolidation performance. A cutting depth of 0.62 mm per revolution was set for gypsum plasters, with an initial porosity of 29% to 38%, showing average values of 0.24–0.82 N mm^−1^ for untreated pieces, up to ~2.4 N mm^−1^ for PEMA/PMA coating (~3 mm depth), up to ~1.25 N mm^−1^ for PVB coating (~2 mm depth), and ~0.8 N mm^−1^ all over the depth for TEOS coating (see Figure 7a). For the bioconsolidation treatment, ~1.7 N mm^−1^ was obtained throughout the whole depth of the treated piece. Moreover, the bacterial treatment decreased gypsum porosity from 48% to 41%, increasing average DR from 0.24 N mm^−1^ up to 1.38 N mm^−1^ at ~1.5 mm depth, with a strengthening effect extended ~6 mm depth (see Figure 7b). SEM micrographs and XRD showed newly precipitated spherulites of vaterite, with an average crystallite size of ~23 nm. These spherulites were surrounded by bacterial EPS and calcified bacterial cells without pore plugging. Cross-section analysis showed bacterial precipitates homogeneously distributed in the samples. SEM images for the conventional consolidant coatings showed the formation of a surface film, which blocked the pores (see Figure 7c–f). Colorimetry analysis revealed total color change values (∆E) of ca. 5 for almost all the treated samples, which is an acceptable value according to conservation guidelines.

Bucci et al. reported MICP treatment for an artificial fractured sandstone core specimen with a porosity of 12.25% [77]. The strategy carried out by the researchers involved the use of the ureolytic bacteria *Sporosarcina pasteurii*. Sandstone was saturated with deionized water for about 24 h. Bacterial incubation was performed in urea and CaCl_2_ containing media (pH ~ 6). Treatment was divided into various separate injections of ~25 mL of inoculated and sterile media in the saturated sandstone core. Between each injection, a period of 24 h was left to accomplish bacteria surface attachment and calcite precipitation. Results showed that fracture permeability was decreased by ~29%. Besides confirmed visual precipitation, X-Ray CT allowed the comparison of three cross-sections of the core, showing effective sealing of the rock fracture.

**Figure 8:**
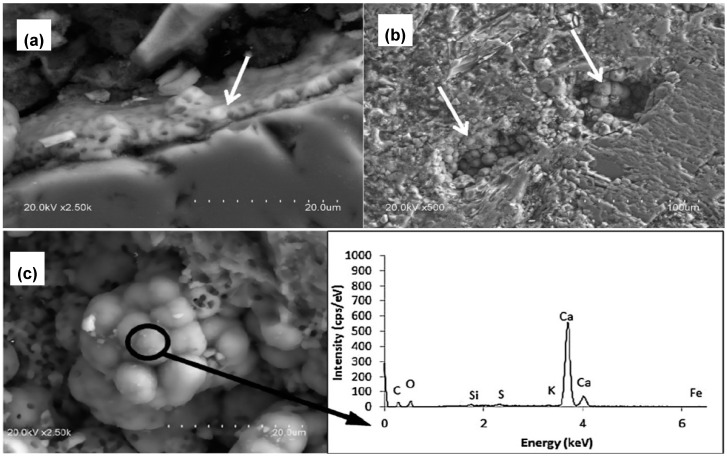
SEM images of (**a**) CaCO_3_ precipitated over recycled aggregates surface, (**b**) CaCO_3_ precipitated inside a pore of recycled aggregates (RA) sample, (**c**) CaCO_3_ globular precipitate with Energy-dispersive X-ray spectroscopy (EDX) spectrum showing the composition of the precipitate. Adapted with permission from [78] 2017, Elsevier.

Quality improvements of mixed and ceramic recycled aggregates (RAs) obtained from construction demolition waste were studied by García-González et al. using MICP treatments [78]. Ceramic RAs from different recycling plants were specially chosen by their ceramic composition as follows: TEC-REC (Tecnología y Reciclado S.L., Madrid-Spain, 33.6% ceramic), ANTWERP (Antwerp Recycling Company, Antwerp-Belgium, 38.4% ceramic), and BIERZO (Bierzo Recicla S.L., Leon-Spain, 97.9% ceramic) samples. *L. sphaericus* LMG 222 57 was used as carbonatogenic bacteria. Biodeposition liquid culture media consisted of yeast extract, urea, and calcium nitrate. After cleaning RA samples in HCl and distilled water, samples were submerged for 24 h in a liquid culture of *L. sphaericus* under non-sterile and static conditions at 20 °C. Then, RA samples were submerged in the biodeposition liquid media for four days. Results showed weight gain between 16% and 46% for small samples (4–12.5 mm) in comparison to the large samples (12.5–20 mm). Samples with increased ceramic content gained more weight, which is attributed to greater roughness of the ceramic surface. Moreover, continuous calcium carbonate layers were present in less irregular ceramic surfaces. Water permeability reduction was observed in all tested samples. Comparison between small and large samples showed a difference in water permeability of 46%, 41%, and 16% for TEC-REC, ANTWERP, and BIERZO, respectively. Samples’ resistance against ultrasonic attack exhibited different responses between biotreated and untreated samples. TEC-REC large samples registered 44% weight loss and small samples 6% weight loss. For samples with higher ceramic content, biotreated samples exhibited higher weight loss than untreated samples. SEM analysis revealed calcium carbonate uniform deposition over a regular surface and partial pore surface filling by globular precipitates (see Figure 8a,b). EDX revealed that globular precipitates were likely to be calcium carbonate (see Figure 8c).

**Figure 9:**
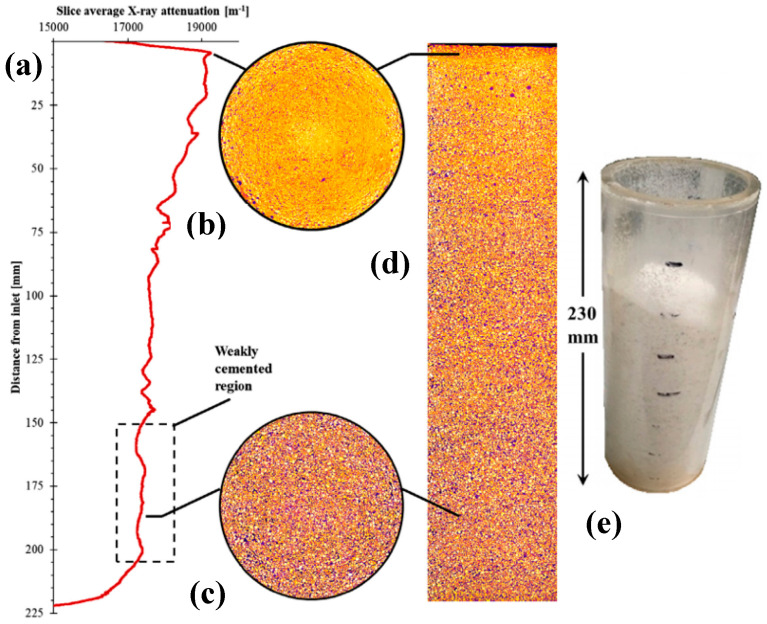
X-ray computed tomography (X-CT) column scans, showing in yellow-white colors high attenuation from solid material. Purple-black colors indicate low attenuation (e.g., air-filled pore space). (**a**) Slice averaged X-ray attenuation vs. distance from inlet column; (**b**) Maximum average attenuation; (**c**) Attenuation from the weakly cemented region; (**d**) Overall vertical profile attenuation; (**e**) Inverted column after non cemented sand removal. Adapted with permission from [79].

Minto et al. studied the restoration of degraded marble structures using MICP by X-ray computed tomography (X-CT) [79,80]. The carbonatogenic bacteria used was *Sporosarcina pasteurii* DSM-33. Biodeposition inducing media was composed of urea and CaCl_2_ at pH = 6.5. Samples of marble were obtained by crushing marble gravel until a particle size of 0.5–1.4 mm and filled into a column. Bacteria culture and biodeposition media were injected every day for six days. After completion of MICP treatment, results revealed that the inlet surface had greater cementations compared to outlet marble grains. After each injection, measurements showed a gradual permeability decrease. Porosity was also reduced from 32.4% before MICP treatment to ca. 28% after MICP treatment. At 4.5 mm depth from the inlet, a minimum porosity of 7.2% was obtained. From inlet to outlet, non-heterogeneous precipitation was observed by X-CT, resulting in a color gradient (see Figure 9). Different injection strategies should be applied for in situ restoration to reach more homogeneous precipitation. MICP treatment was able to cement the marble crushed pieces, meaning that the method allows restoration of the considerable size cracks.

Hudyma et al. presented a study of MICP on coquina core specimens [81]. Coquina is a cemented limestone composed of shells and quartz sand. Thus, calcite, phosphate, and siliciclastic material are the main chemical components of coquina [82,83]. Core specimens were cut in ~50 mm diameter from two blocks, labeled as J and K. Treatment began with the immersion of the coquina samples in a *Sporosarcina pasteurii* solution as calcifying bacteria for one hour, allowing saturation and penetration of bacteria in the limestone. Then, coquina samples were immersed in biodeposition media containing an equimolar solution of CaCl_2_ and urea. Treatment with the biodeposition media was performed during 2, 10, 20, and 40 days. For coquina specimen JZ14-P2, evident crystal deposition was observed as a protective surface, without the presence of calcite deposition inside the pores, while specimen JX12-P2 showed spheroidal MICP deposition inside the stone pores. Measurements revealed a global unit weight increase after treatment between 1.1% and 9.7%. Overall, the water absorption decrease was between 2.1% and 47.9% for the different samples. MICP treatment of coquina samples clearly enhanced the protective properties of the material.

**Figure 10:**
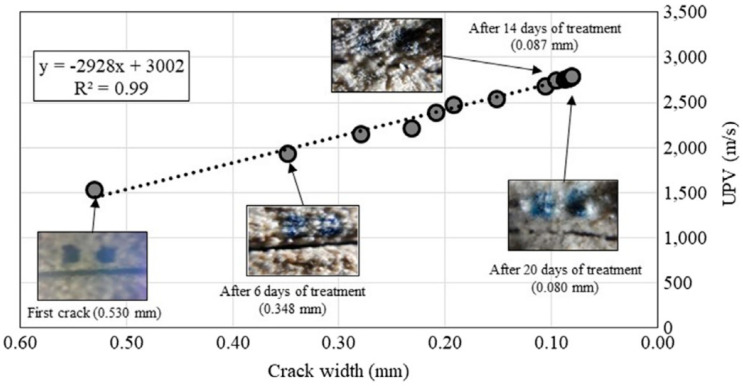
The plot of ultrasonic pulse velocity (UPV) value vs. crack width of MICP-treated sample along 20 days of treatment. Adapted with permission from [84]. 2019, Elsevier.

Jongvivatsakul et al. investigated the crack healing performance within cement mortars using MICP [84]. *L. sphaericus* LMG 2257 was used as calcifying bacteria, while biodeposition media consisted of nutrient broth, CaCl_2_, and sodium bicarbonate (NaHCO_3_) at pH = 8. Mortar samples were prepared from Portland cement. Artificial cracks were prepared by placing a copper plate in fresh samples. Each day inoculated biodeposition media and urea were applied to the mortars for 20 days. MICP treatment was visually evaluated through 40× magnification photographs (see insets, Figure 10). After six days of treatment, crack width was decreased by about 34%; after 16 days, remediation was decreasing up to 84%. Non-significant changes were observed in the last 4 days. Samples were divided into treated and untreated zones. SEM images showed vaterite as the main phase in the treated zone. EDS analyses revealed the presence mostly of calcium, carbon, and oxygen. Qualitative XDR analysis identified the presence of calcite and vaterite in the treated zones. For the first 5 days, artificial crack remained apparently non healed, pointing out for artificial crack healing that began from inside to outside. Ultrasonic pulse velocity (UPV) [85] measurements demonstrated that the pulse velocity increased linearly for the first 14 days in treated samples (see Figure 10). Compressive strength increased from 17.3 MPa (cracked mortar) to 24.7 MPa (healed mortar). Water adsorption was measured, showing ~72% lower adsorption for healed mortars compared to cracked samples. Water penetration was 10.8 mm and 8.6 mm depth in the mortars before and after treatment, respectively. CaCO_3_ formation not only filled the artificial crack but also improved the quality of the mortars. Compressive strength and UPV measurements confirmed that the MICP-treated mortars became stronger materials compared to the original samples.

**Figure 11:**
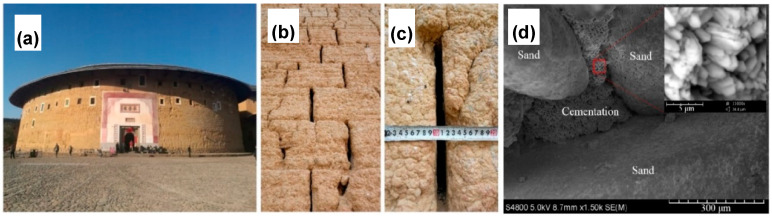
Photographs of (**a**) The King of Tulou – Chengqilou, (**b**) and (**c**) wall deteriorated cracks (width is approximately 1.5 cm, depth is up to 100 cm). (**d**) SEM images of samples after MICP treatment. Adapted with permission from [86] 2020, Elsevier.

Liu et al. studied the protection and restoration of cracks in Tulou or earthen buildings (see Figure 11a) made of mainly tabia [86]. Tabia consists of sand, limestone (CaCO_3_), and clay, which usually possess a low tensile strength. Cracks tend to occur in the wall of the Tulou as starting points for damage propagation (see Figure 11b,c). Soil samples were collected nearby earthen buildings for the preparation of probes. Soil analysis showed the presence of silica and kaolinite. After mixing soil samples with sand and lime, mixtures were compacted in different shapes, such as cylinders, beams, and wallets. Bacterial solution and cementation media were injected every day for three days. *Bacillus pasteurii* (DSM-33) was used as calcifying bacteria, while cementation media was composed of urea and CaCl_2_. Unconfined compressive strength (UCS) tests were carried out for the samples, indicating that mixture samples after treatment increased the average UCS. SEM images revealed that MICP treatment was able to bind loose soil particles (see Figure 11d). XRD analysis showed the presence of calcite, as the CaCO_3_ unique phase, and SiO_2_ peaks from the soil. Flexural strength (FS) was measured before and after MICP treatment to determine the recovery ratio of FS. The repair rate of FS average recovery measurements indicated 35.2%, 56.86%, and 79.92% for crack widths of 15 mm, 10 mm, and 5 mm, respectively. The recovery ratio of shear strength after MICP treatment was 50.74%, 69.53%, and 88.54% for crack widths of 15 mm, 10 mm, and 5mm, respectively. Static contact angle after MICP treatment was found between 83.6° and 100° compared to a contact angle 0° for untreated samples, pointing out for an increased hydrophobicity of the tabia samples.

**Figure 12:**
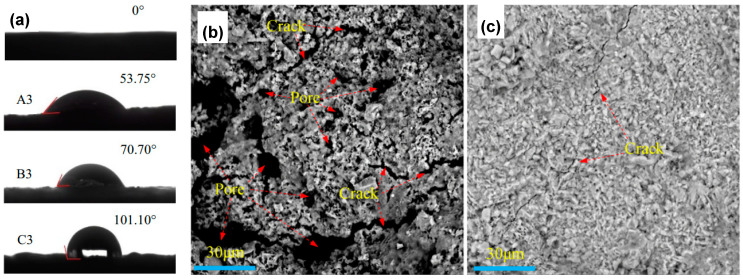
(**a**) Water static contact angles of samples before (0°) and after treatment (A3, B3, C3). SEM images of ancient tiles (**b**) before and (**c**) after MICP treatments. Adapted with permission from [87] 2020, Elsevier.

Liu et al. studied the formation of an antierosion layer by means of MICP to reduce the weathering caused by rain erosion on ancient clay roof tiles [87]. Clay samples were obtained from Shaoming Lou, a Chinese Hakka Tulou, built in 1915 and located in Longyan city. Calcifying bacteria used were *Bacillus pasteurii* DSM 33. Consolidation media consisted of calcium chloride and urea. Clay samples were cleaned and cut into 5 cm diameter discs. Bacterial solution and consolidation media were brushed over the sample surface three times. After brushing, samples were left in static conditions at 30 °C for 7 days. Measurements of static contact angle of water showed contact angles up to 101°, indicating an increased hydrophobicity of sample surface treated with MICP (see Figure 12a). SEM micrographs showed different imperfections in the surface of the original samples (see Figure 12b), which were almost completely disappeared after MICP treatment (see Figure 12c). The width of cracks was significantly reduced, preserving the air permeability. EDS analysis of precipitated crystals indicated as main elements Ca, O, and C, supporting the presence of CaCO_3_. The thickness of the MICP surface layer increased with increasing concentration of the bacterial solution and consolidation media. Capillary water absorption was reduced in all treated samples; however, higher concentrations of bacteria solution (10^8^ cell/mL) had poorer contributions, reducing water absorption. Coatings were resistant to acid corrosion tests, whose pH was higher than 1.0. The total color difference showed low values (∆E < 3), indicating that the MICP treatment met standard color conditions.

## 5. Microbiologically Induced Carbonate Precipitation for In Situ Restoration of Historical Structures

Métayer-Levrel et al. went further with induced bacterial precipitation by testing over miniature walls. Limestones used in this study were Tuffeau (small pores), Saint-Maximin, and Saint-Vast (large pores) [88]. Patented bacteria and culture media met industrial and financial requirements. Treatment media inoculated with a bacterial strain was sprayed over the limestone surface, followed by spraying of sterile media every 24 h for 5 days and then every 48 h for 10 days. After treatment, a biocalcin coating of several micrometers was observed. Mineral particles filled the voids moderately by getting engrained in the structure. In situ tests were carried out on the SE tower of Saint Médard Church, located in Thouars, France. Tuffeau limestone was used to build the church. Treatment began in June 1993 over an area of 50 m^2^. The bioprecipitated produce was exposed to normal weather variations. Treatment effects were evaluated after 6 months and 1 year by measuring surficial permeability (a measurement of the standard time of water absorption using a water pipe). MICP treatment increased the time of water absorption from ~70 s (before treatment) to ~250 s after 6 months of treatment but then decreased to ~130 s after one year of treatment. In any case, the surficial permeability after treatment showed lower values if compared to before treatment values. No appreciable changes for gas permeability, color, or aesthetic appreciation were observed if compared to the original samples. Bioprecipitate guaranteed the preservation of the limestone by reducing the exchange of degrading agents and environmental pollutants. Moreover, calcifying bacteria bulk population interfered with the development of acidifying bacteria, which might be a threat to historical buildings. Finally, no changes in the external aspect of the tower were observed after 3.5 years.

Perito et al. reported a bacterial CaCO_3_ mineralization treatment on the Andera Cathedral, Italy [14]. First, stone samples (10 cm × 10 cm × 4 cm) obtained from the main façade of the Pietra d’Angera lithotype were tested in the laboratory. *B. subtilis* 168 strain was the calcifying bacteria, and B4 was used as the precipitation medium. After bacterial incubation in B4 media at 37 °C, cells were collected at a 10^8^ cells/mL concentration, centrifugated, autoclaved, stripped of metal cations through deionized water, rinsed, and stored in physiological solution (PS) at −20 °C. After dilutions of PS in calcium chloride solution, ammonium carbonate vapors induced the precipitation of calcium carbonate. Besides crystallization, no visible changes were observed after the seventh day at room temperature. Then, the frozen cell mixture was ground with alumina, then unbroken cells and alumina were eliminated through centrifugation. Cytosol, membranes, and cell walls were stored at −20 °C and labeled as *Bacillus* cell fraction (BCF). Finally, BCF was lyophilized and resuspended in CaCl_2_ solution mixed with supersaturated calcium bicarbonate solution (Super C) and enriched with 20 nm calcite nanoparticles (2% *w/v*). Around 30 mL per application was sprayed on the stone samples surface twice a day for 3 days. A stone sample was sprayed only on Super C as reference. In vitro tests showed an overall color change ∆E < 3, meaning acceptable and no detectable changes. Water absorption decreased by 16.7%. Cohesion profiles from DR did not show remarkable differences. In situ test was implemented on the main façade of the Angera Cathedral, which is dated from the sixth century. Selected areas sized 0.29 m^2^ and 0.28 m^2^, with a 0.04 m^2^ area as a control. Approximately 1 L m^−2^ per spray application of BCF (8.5 g L^−1^) in Super C solution and bacteria-free super C solution was sprayed for the first day. BCF (0.032 g L^−1^) in Super C, enriched with nanoparticles, and bacteria-free Super C were sprayed for the second day. On the third day, only a bacteria-free Super C enriched solution was sprayed on the chosen areas. After four months, evaluation tests were performed, such as water absorption, surface color change, and cohesion profiles. Small cores from the chosen areas were taken to the laboratory to analyze new calcite penetration. CaCO_3_ precipitated crystals were collected and analyzed with FT-IR and XRD, indicating the presence of calcite. In situ color test measurements showed ∆E < 3, meaning no detectable changes to the human eye. Water absorption decreased by 6.8% for in situ experiments. Cohesion profiles indicated an increased hardness of the treated areas for the first 3 mm.

Rodriguez-Navarro et al. reported in situ restoration using MICP over stone monuments dated from the sixteen century in Granada, Spain [4]. Heavily degraded areas from three different test sites were treated: (i) Hospital Real, (ii) San Jeronimo’s Monastery Apse, and (iii) Capilla Real. These three buildings were constructed using porous calcarenite, which is a material with high water absorption and porosity. Treatments were performed with M-3P medium inoculated with *M. xanthus*. The inoculated medium was sprayed twice on the treated area, followed by one spray of sterile medium. Treatment was applied twice a day for six consecutive days. After each application, the treated area was wrapped with aluminum/plastic foil to avoid possible pigmentation by sunlight effect and to reduce media evaporation. The binding capacity of treatments, surface strengthening, and chromatic changes were tested for four years after initial treatment. Peeling tape test showed weight loss reduction after inoculated media treatment for all the treated areas, which is related to the bacterial CaCO_3_ precipitation (calcite and vaterite, according to XDR). SEM analysis showed that new crystals were precipitated aligned to the pore system without plugging them. Moreover, loose calcarenite grains were connected via EPS with new bacterial calcium carbonate. Results with sterile M-3P media showed an identical degree of consolidation for San Jeronimo and Hospital Real treated areas. In some cases, sufficient consolidation was obtained with the activation of the original carbonatogenic bacteria present, thus reducing treatment cost. Moreover, spectrophotometric ∆E measurements were below 5, meaning acceptable value for treatment.

**Figure 13:**
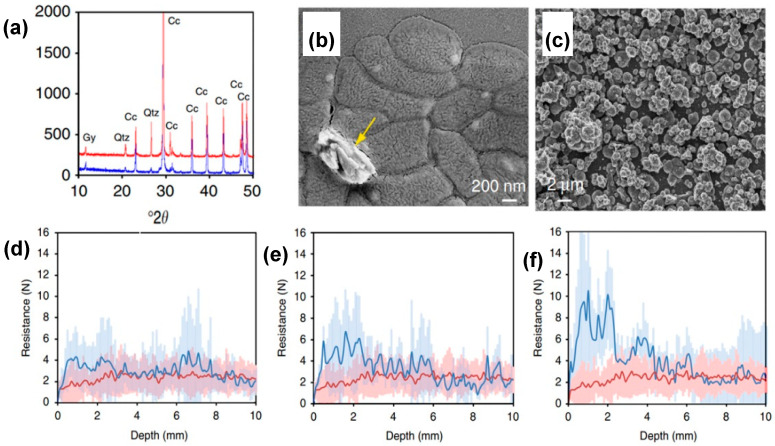
(**a**) XRD pattern before (blue) and after (red) MICP treatment. SEM images of (**b**) newly formed CaCO_3_; (**c**) calcite substrate covered by bacterial calcite. Drilling resistance depth profile: (**d**) *M. xanthus* treatment (blue-line); (**e**) M-3P treatment (blue-line); (**f**) self-inoculated treatment (blue line). Untreated stones (red line) are presented for comparison. Shaded areas represent s.d. (±1σ). Adapted with permission from [89].

Self-inoculation of indigenous calcifying bacteria present in stones prior to MICP treatment is another viable way that Jroundi et al. designed for in situ treatment of salt weathered stones [89]. The cloister entrance of San Jeronimo Monastery, Spain, presented granular disintegration and surface loss. This weathering and erosion were attributed to the crystallization of salts, such as magnesium sulfate hexahydrate $\left({\mathrm{MgSO}}_{4}\cdot 6H_{2}O\right)$, niter (KNO_3_), halite (NaCl), among others. Fifty-five bacterial isolates were obtained from selected areas. Incubation of M-3P inoculated medium led to high CaCO_3_ precipitation in all bacterial isolates after 48 h. XDR analysis identified calcite as the main phase and <10 wt% of vaterite. To evaluate the applicability of the activated carbonatogenic bacteria, the sterile calcite substrate was immersed in an inoculated M3-P medium. Field emission scanning electron microscopy (FESEM) [90] and transmission scanning electron microscopy (TSEM) [91] showed dense bacterial colony formation and CaCO_3_ embedded in EPS after 20 h treatment (see Figure 13b). Spheroidal or rhombohedral calcite structures fully covered the calcite substrate growth controlled on a self-epitaxy way after 48 h treatment (see Figure 13c). In situ treatments were applied on stone blocks with a similar degree of exposure and decay. Three different treatment approaches were performed using *M. xanthus*, sterile M-3P media, and a self-inoculation biotreatment. *M. xanthus* and sterile M-3P treatment results showed limited consolidation. The peeling tape test revealed modest surface consolidation for both treatments after 5 months. Drilling resistance values were slightly higher in the first ~5 mm depth for treated areas after 24 months. SEM showed almost no presence of calcium carbonate or EPS. Chromatic changes revealed, for both treatments, the acceptable values of ∆E (<5). On the other hand, self-inoculated bacteria treatment showed remarkable consolidation by a peeling tape test along with a 24 months study. DR measurements showed about 4 times higher values than untreated stone (see Figure 13d–f). The maximum DR value achieved was 10 ± 6 N for the first 3 ~ 5 mm depth. Color changes after treatment were not significant, showing an ∆E = 3.8 ± 1.7. XRD analyses of samples from treated areas showed calcite as the unique calcium carbonate phase (see Figure 13a). Strengthening of the stone was associated with the abundant presence of newly precipitated calcium carbonate crystals and EPS. Mercury intrusion porosimetry (MIP) showed a reduction in porosity from 27 ± 1% to 25 ± 1%, resulting from the formation of 30–100 nm size nanocrystals that cemented the stone without plugging the pores. No presence of material loss or granular disaggregation was observed 24 months after the treatment.

## 6. Conclusions and Perspectives

Highly ureolytic bacteria are the main strains of microorganisms used in MICP-treatment for the restoration of historic buildings. Despite higher calcium carbonate production by some microorganisms, the usage of human pathogenic bacterial strains (e.g., *P. putida*, *B. cereus)* is not recommended because restoration in situ lacks the biosafety that a microbiology laboratory could provide. MICP treatment has been applied in different ways, such as immersion, spraying, injection, and brushing. This is an important parameter, necessarily to be taken, depending on the in-situ circumstances and requirements. Optimization of the treatment, such as lowering reagents costs, high yields of carbonate production, shorter treatment time, avoiding unnecessary tests or treatment steps, could allow researchers to save some time, money, and resources.

Although, until now, the method has not been standardized, a good approach for this is the usage of the ζ-potential because it has been proven that bacteria with lower ζ-potential possess higher surface colonization capacity. A modification in the MICP treatment to obtain CaCO_3_ crystals using a dead cell fraction instead of the application of live calcifying bacteria becomes interesting for the case in which certain bacterial strains, like nitrifying or sulfur-oxidizing bacteria, are needed, e.g., *Nitrosomonas spp*, *Nitrobacter spp*, *Thiobacillus spp*. These bacteria species are capable of excreting acids [92] (if nitric acid is produced, it can react with calcite, thus forming highly soluble calcium nitrate), leading to biocorrosion of the material [23]. These problems were addressed by Ganendra et al. [93], using *Methylocystis parvus* as calcifying bacteria to induce calcium carbonate precipitation from different calcium formate concentrations. Although culture-dependent methods have been useful to isolate carbonatogenic microorganisms, new genomic microbiological analyses have allowed us to study the epilithic microbial biodiversity in monuments and historical buildings [94]. For example, Chimenti et al. carried out a metagenomic analysis of the bacterial communities, colonizing the walls of the medieval church of San Leonardo di Sponto, in Italy [95]. The microbiome of the wall samples was composed not only of possible deteriorated microorganisms but also carbonatogenic bacteria corresponding to the phylum Actinobacteria, specially *Arthrobacter* spp. In fact, in brick samples, this phylum may be predominant, depending on the environmental conditions [96]. DNA next-generation sequencing studies have focused on the microbial diversity in bricks of the former Auschwitz II-Birkenau concentration, and extermination camp in Poland has shown the presence of the genus *Arthrobacter* as part of the microbiota [97]. Although these microorganisms can colonize these surfaces, the carbonatogenic bacteria distribution may differ in relation to the collection site. For instance, Andrei et al. found that the diversity of epilithic bacterial communities on Saint Donatus statue, Romania, varied depending on the monument sampling site [98]. Microbiologically induced carbonate precipitation has been shown to be an efficient strategy to consolidate, protect, restore, and enhance the mechanical properties of a broad variety of materials used in historical buildings.

## Abbreviations

AB-PAS	Alcian blue-periodic acid-Schiff stain
BCF	Bacillus cell fraction
CTC	5-Cyano-2,3-ditolyl tetrazolium chloride
DR	Drilling resistance
EDX	Energy-dispersive X-ray spectroscopy
EPS	Extracellular polymeric substances
ESEM	Environmental scanning electron microscopy
FESEM	Field emission scanning electron microscopy
FS	Flexural strength
MICP	Microbiologically induced carbonate precipitation
MIP	Mercury intrusion porosimetry
PEMA/PMA	Poly(ethyl methacrylate-co-methacrylate)
PS	Physiological solution
PVB	Polyvinyl butyral
RA	Recycled aggregates
SEM	Scanning electron microscopy
Super C	Supersaturated calcium bicarbonate solution
TEOS	Tetraethyl orthosilicate
TSEM	Transmission scanning electron microscopy
UCS	Unconfined compressive strength
UPV	Ultrasonic pulse velocity
X-CT	X-ray computed tomography
XRD	X-ray powder diffraction
ζ-potential	Electrical charge at the shear plane
ΔE	Overall degree color change
